# Polychlorinated Biphenyl (PCB) Exposure and Diabetes: Results from the Anniston Community Health Survey

**DOI:** 10.1289/ehp.1104247

**Published:** 2012-02-14

**Authors:** Allen E. Silverstone, Paula F. Rosenbaum, Ruth S. Weinstock, Scott M. Bartell, Herman R. Foushee, Christie Shelton, Marian Pavuk, for the Anniston Environmental Health Research Consortium

**Affiliations:** 1 State University of New York Upstate Medical University, Syracuse, New York, USA; 2 Department of Veterans Affairs Medical Center, Syracuse, New York, USA; 3 University of California–Irvine, Irvine, California, USA; 4 University of Alabama–Birmingham, Birmingham, Alabama, USA; 5 Jacksonville State University, College of Nursing and Health Sciences, Jacksonville, Alabama, USA; 6 Agency for Toxic Substances and Disease Registry, Atlanta, Georgia, USA

**Keywords:** diabetes, epidemiology, polychlorinated biphenyls (PCBs), POPs

## Abstract

Background: Polychlorinated biphenyls (PCBs) manufactured in Anniston, Alabama, from 1929 to 1971 caused significant environmental contamination. The Anniston population remains one of the most highly exposed in the world.

Objectives: Reports of increased diabetes in PCB-exposed populations led us to examine possible associations in Anniston residents.

Methods: Volunteers (*n* = 774) from a cross-sectional study of randomly selected households and adults who completed the Anniston Community Health Survey also underwent measurements of height, weight, fasting glucose, lipid, and PCB congener levels and verification of medications. Odds ratios (ORs) and 95% confidence intervals (CIs) were calculated to assess the relationships between PCBs and diabetes, adjusting for diabetes risk factors. Participants with prediabetes were excluded from the logistic regression analyses.

Results: Participants were 47% African American, 70% female, with a mean age of 54.8 years. The prevalence of diabetes was 27% in the study population, corresponding to an estimated prevalence of 16% for Anniston overall; the PCB body burden of 35 major congeners ranged from 0.11 to 170.42 ppb, wet weight. The adjusted OR comparing the prevalence of diabetes in the fifth versus first quintile of serum PCB was 2.78 (95% CI: 1.00, 7.73), with similar associations estimated for second through fourth quintiles. In participants < 55 years of age, the adjusted OR for diabetes for the highest versus lowest quintile was 4.78 (95% CI: 1.11, 20.6), whereas in those ≥ 55 years of age, we observed no significant associations with PCBs. Elevated diabetes prevalence was observed with a 1 SD increase in log PCB levels in women (OR = 1.52; 95% CI: 1.01, 2.28); a decreased prevalence was observed in men (OR = 0.68; 95% CI: 0.33, 1.41).

Conclusions: We observed significant associations between elevated PCB levels and diabetes mostly due to associations in women and in individuals < 55 years of age.

Diabetes incidence is increasing worldwide. Increasing obesity and sedentary lifestyle are major risk factors, but other environmental factors may also be important. Toxicants such as polychlorinated biphenyls (PCBs) and dioxins are postulated to play a role, based on data from the National Health and Nutrition Examination Survey (NHANES) (Lee et al. 2006, 2007) and studies from the Slovak Republic (Langer et al. 2002; Radikova et al. 2004), Sweden (Lee et al. 2011a), Japan (Tanaka et al. 2011; Uemura et al. 2008), Taiwan (Wang et al. 2008), and the United States (Codru et al. 2007; Lee et al. 2010, 2011b; Persky et al. 2011; Turyk et al. 2009a, 2009b; Vasiliu et al. 2006), but the results are inconsistent. In addition, commentaries (Jones et al. 2008; Porta 2006) have emphasized the need for additional research.

PCBs were produced in Anniston, Alabama, from 1929 to 1971 in a plant that was purchased by the Monsanto Corporation in 1935. Large amounts of PCB-containing waste discharged during production were revealed during litigation in Anniston (Grunwald 2002), with widespread contamination of soil, sediment, and air still found in the late 1990s [Agency for Toxic Substances and Disease Registry (ATSDR) 2000]. Evaluation of PCB levels in sera from adults in Anniston collected from 1996 to 1999 in smaller nonsystematic studies, or as a part of litigation, indicated that this was one of the most highly exposed communities in the world (ATSDR 2000; Hansen et al. 2003). In 2003, the ATSDR awarded funding to the Anniston Environmental Health Research Consortium (AEHRC) to conduct a health survey in Anniston adults. In this study, we examined the prevalence of diabetes in this population and the association of their serum PCB levels with diabetes.

## Methods

*Study population.* The Anniston Community Health Survey employed a two-stage sampling procedure in which 3,320 households were randomly selected from a commercial list of all residential sites within the city limits. Addresses in west Anniston, the location of the former PCB manufacturing facility, were oversampled. All sampled addresses were visited by study staff: 489 were found to be vacant or nonresidential, and residents of 890 could not be contacted after multiple attempts. Contact was made with a member of each of the remaining 1,823 targeted households, and 713 declined to participate. Among the remaining 1,110 consenting households, an adult > 18 years of age was randomly selected for survey completion, resulting in an overall participation rate of 61% (1,110/1,823). The study was reviewed and approved by the University of Alabama–Birmingham’s Institutional Review Board, and all participants provided written informed consent.

*Data collection.* The Anniston Community Health Survey included a 45-page questionnaire developed by the AEHRC. Demographic information, medical and family history, and health behaviors were recorded. The survey was administered by trained interviewers at the homes of participants (October 2005–April 2007). The length of the interview was typically 45–60 min.

Of the 1,110 individuals who completed the survey, 774 agreed to a clinic visit, which included measurements—by standard protocol—of height, weight, waist circumference, and blood pressure, as well as a review of current medications. Fasting blood was obtained for analyses of glucose and lipids (Jacksonville Medical Center, Jacksonville, AL), the major 35 *ortho*-substituted PCB congeners, and 13 pesticides and herbicides [Division of Laboratory Sciences at the Centers for Disease Control and Prevention’s (CDC) National Center for Environmental Health, Atlanta, GA]. The PCB congeners and pesticides were measured in serum using high-resolution gas chromatography/isotope-dilution high-resolution mass spectrometry (SjÖdin et al. 2004). Serum total lipids were calculated with the enzymatic summation method using triglyceride and total cholesterol measurements (Bernert et al. 2007).

Total wet-weight levels of the 35 PCB congeners in nanograms per gram (parts per billion) were summed (ΣPCBs). Before summation, values below the limit of detection (LOD) were substituted with the congener-specific LOD divided by the square root of 2. The ΣPCB variable was divided into quintiles, with the first quintile serving as the referent category. Quintile limits were based on values from the entire group with PCB measurements. Because the PCB distribution showed a positive skew, a log-transformed version of the continuous wet-weight total PCB variable was derived for trend assessment and for use in subgroup analyses. Consistent with recommendations from Schisterman et al. (2005), we evaluated both wet-weight PCB totals (nanograms per gram), with control for total lipids, and lipid-adjusted PCB values, but we report only the former here. Subsets of PCBs based on structure and function were summed (log-transformed) as follows: estrogenic congeners 44, 49, 66, 74, 99, 110, and 128 (DeCastro et al. 2006); mono-*ortho* congeners 28, 66, 74, 105, 118, 156, 157, 167, and 189; mono-*ortho* dioxin toxic equivalents (TEQ) 105, 118, 156, 157, 167, and 189 (Van den Berg et al. 2006); di-, tri-, and tetra-*ortho* congeners (combined) 44, 49, 52, 87, 99, 101, 110, 128, 138+158, 146, 153, 170, 172, 180, 194, 149, 151, 177, 178, 183, 187, 195, 196+203, 199, 206, and 209; and the ryanodine-like congeners 52, 101, 149, 151, 170, 180, 183, and 187 (activate ryanodine receptors at < 1 μM) (Pessah et al. 2006). In addition, we evaluated the possible association between log-transformed dichlorodiphenyldichloroethylene (DDE) and diabetes.

For this study, diabetes was defined as self-report of physician-diagnosed diabetes or fasting glucose > 125 mg/dL. Prediabetes was defined as fasting glucose 100–125 mg/dL, absence of previously diagnosed diabetes, and absence of glycemic control medications. Normoglycemia was defined as individuals with a negative diabetes self-report, fasting glucose < 100 mg/dL, and the absence of glycemic control medications. Ever-smoking was defined as having smoked ≥ 100 cigarettes over a lifetime. A positive family history of diabetes was defined as the presence of diabetes in siblings, parents, grandparents, aunts, or uncles. Participants were Caucasian (53%), African American (46%), and Native American (1%) and were grouped as white and nonwhite (African Americans, Native Americans) for purposes of these analyses.

Laboratory results [glucose, total cholesterol, high-density lipoprotein (HDL), low-density lipoprotein (LDL), triglycerides, PCB, and pesticide levels] were available for 765 of the 774 participants. Demographic information was missing for < 3% of participants, with the exception of self-reported income (missing for 28%). Because individuals with missing data were excluded from the analyses, income was not included as a measure of socioeconomic status in multivariable models.

*Statistical analyses.* Preliminary analyses included the calculation of frequencies, means, and SDs and the use of *t*-tests, one-way analysis of variance, general linear models (GLMs), and Pearson’s chi-square test. A GLM was used to calculate geometric means (GMs) using the antilog of the log-transformed PCB mean value adjusted for age, sex, race/ethnicity, or body mass index (BMI) (Bland and Altman 1996). Unconditional logistic regression modeling compared participants with and without diabetes by quintiles of the ΣPCBs. Participants were excluded from multivariable analyses if they had prediabetes (*n* = 171) because many of their disease and demographic characteristics were intermediate to those observed in individuals with diabetes and those with normoglycemia. Associations between PCB levels and established diabetes risk factors (BMI, family history of diabetes, age, race/ethnicity), sex, other possible confounders (i.e., education, marital status, smoking, duration of residence in Anniston), and diabetes were assessed through bivariate logistic regression analysis. Confounding was assessed in a second series of models that included PCBs and one of the established diabetes risk factors or potential confounders. Confounding was defined as a > 10% change in the β-coefficient and was evaluated by comparing point estimates for the PCB–diabetes associations with and without the potential confounder. Final multivariable models included PCB levels (quintiles or log transformed), age (continuous), sex (female/male), race/ethnicity (white/nonwhite), family history of diabetes (no/yes), BMI (continuous or categorical: ≤ 24.9, 25.0–29.9, 30.0–34.9, 35.0–39.9, ≥ 40 kg/m^2^), total lipids (continuous), and taking lipid-lowering drugs (no/yes).

Stratified analyses were performed by sex, race/ethnicity (white and nonwhite), BMI (≤ 24.9, 25.0–29.9, 30.0–39.9, ≥ 40 kg/m^2^), and age (< 55 and ≥ 55 years of age) because our initial data showed a striking number of younger adults with diabetes. The median age of the study population (55 years) was chosen as the cut point in the age-stratified analyses to have relatively equal sample sizes. The analytic strategy employed in the stratified analyses was similar to that for the entire group of participants. Effect modification was assessed in full models using a backward stepwise procedure and the likelihood ratio test; a *p*-value of ≤ 0.05 indicated the presence of effect modification.

Initial analyses were conducted using quintiles of the ΣPCB variable followed by analyses using the log-transformed wet-weight PCB variable to assess trend; odds ratios (ORs) associated with the log-transformed variables (ΣPCBs, PCB subsets, and DDE) were reported as a 1 SD increase. A generalized additive model (GAM) (Hastie and Tibshirani 1990) was fitted using a cubic regression spline for the ΣPCBs to further examine the shape of the dose–response curve. Analyses evaluating subsets of PCBs (e.g., mono-*ortho*) and DDE were fitted using log-transformed versions of these variables. Covariables included in the GAM and the adjusted PCB subset models were identical to those used in the ΣPCB model. All final models report ORs and 95% confidence intervals (CIs). Analyses were conducted using SPSS (version 19; IBM, Chicago, IL) and R software (version 2.10.1; R Development Core Team 2010).

Log binomial models were fitted to assess the overall association between PCBs and diabetes because the prevalence of diabetes is 27%; ORs are more difficult to interpret than relative risks when the prevalence of the outcome is > 10–20% because they overestimate the effects. These binomial models failed to converge; consequently, in this article we present logistic regression results.

## Results

Participant characteristics are shown in Table 1. Nonwhites (47%) were African American except for four Native Americans. The mean age of participants was 55 years (range, 18–93 years). Approximately 30% of the group had less than a high school education, and 40% were high school graduates with no college. The mean duration of residence in Anniston among participants was 28.8 years, with a range of 0.5–79 years. At the time of data collection, 85% of participants had lived in Anniston for ≥ 5 years, and 11% were lifetime residents.

**Table 1:** Participant characteristics and diabetes status: Anniston Community Health Survey.

Diabetes status				
Characteristic	Normoglycemia (n = 396)	Prediabetes (n = 171)	Diabetes (n = 207)	Total (n = 774)
Age (years)*		51.4 ± 16.8		57.6 ± 13.9		58.7 ± 14.0		54.8 ± 15.9
No. of years in Anniston*		27.3 ± 19.3		29.1 ± 18.5		31.5 ± 19.9		28.8 ± 19.3
Total lipids (mg/dL)*		613.6 ± 138.3		671.6 ± 182.3		639.1 ± 158.3		633.3 ± 155.9
Sex*								
Female		287 (72)		100 (59)		154 (74)		541 (70)
Male		109 (28)		71 (41)		53 (26)		233 (30)
Race/ethnicitya*								
White		226 (57)		88 (52)		97 (47)		411 (53)
Nonwhite		170 (43)		83 (48)		110 (53)		363 (47)
Age (years)*								
18–34		69 (18)		10 (6)		11 (5)		90 (12)
35–44		67 (17)		20 (12)		20 (10)		107 (14)
45–54		84 (21)		39 (23)		47 (23)		170 (22)
55–64		82 (20)		42 (25)		50 (24)		174 (22)
65–74		56 (14)		43 (25)		53 (26)		152 (20)
≥ 75		38 (10)		17 (9)		26 (12)		81 (10)
BMI (kg/m2)*								
< 18.5		5 (1)		0 (0)		0 (0)		5 (< 1)
18.5–24.9		122 (31)		18 (10)		25 (12)		165 (21)
25.0–29.9		106 (27)		56 (33)		43 (21)		205 (27)
30.0–34.9		87 (22)		52 (31)		52 (25)		191 (25)
35.0–39.9		39 (10)		26 (15)		43 (21)		108 (14)
≥ 40		36 (9)		18 (11)		44 (21)		98 (13)
Education								
Through grade 8		37 (9)		14 (8)		25 (12)		76 (10)
Some high school		76 (20)		36 (21)		50 (24)		162 (21)
High school graduate		157 (40)		73 (43)		79 (38)		309 (40)
Some college		85 (22)		33 (19)		45 (22)		163 (21)
College graduate		36 (9)		14 (8)		8 (4)		58 (8)
Smoker								
Never		185 (47)		70 (41)		96 (47)		351 (45)
Ever		211 (53)		100 (59)		110 (53)		421 (55)
Family history of diabetes*								
No		165 (43)		62 (38)		53 (26)		280 (37)
Yes		219 (57)		103 (62)		151 (74)		473 (63)
Taking lipid-lowering medications*								
No		339 (86)		128 (75)		120 (58)		587 (76)
Yes		57 (14)		43 (25)		87 (42)		187 (24)
Self-reported heart diseaseb*								
No		316 (80)		121 (71)		123 (60)		560 (73)
Yes		77 (20)		49 (29)		81 (40)		207 (27)
Data are n (%) or mean ± SD. aOf the 363 nonwhites, 359 were African American and 4 were Native American. bAny self-reported heart disease includes a history of myocardial infarction, congestive heart failure or other heart disease. *p ≤ 0.05 in one-way ANOVA (means) or Pearson chi-square test, across all diabetes status categories.								

Diabetes was present in 27% of 774 participants (self-report, *n* = 177; negative self-report and fasting glucose values > 125 mg/dL, *n* = 30). Seventy-five percent of individuals self-reporting diabetes were taking glycemic control medications (data not shown). On average, participants classified as having diabetes or prediabetes were older than those classified as normoglycemic (Table 1). The prevalences of prediabetes and diabetes were greatest in those between 45 and 74 years of age. The ratio of white to nonwhite participants was 1.3 in those without diabetes and 0.89 in those with diabetes (*p* = 0.05), suggesting a greater prevalence of diabetes in the nonwhite population. A greater proportion of participants with diabetes had BMIs ≥ 35 kg/m^2^, positive family histories of diabetes, and heart disease.

Relationships between PCB levels and diabetes status are shown in Tables 2 and 3. Participants with diabetes had significantly higher age-adjusted GM PCB levels than did those with prediabetes or normoglycemia (Table 2); age-adjusted arithmetic means did not differ significantly by diabetes status. Age-adjusted GM PCB levels also were significantly higher for both females and males classed as having diabetes compared with those with prediabetes or normoglycemia. Although mean PCB levels for whites and nonwhites were higher in those with diabetes than in those without diabetes, the differences were not statistically significant. GM PCB levels were similar for males and females, whereas nonwhites had significantly higher PCB levels than did whites. In general, PCB levels were higher in participants ≥ 55 years of age than in those < 55 years of age (Table 3). Within specific age strata, PCB levels remained similar across BMI categories for those ≥ 55 years of age. PCB levels showed inverse associations with BMI in younger individuals with diabetes, and in those with prediabetes, although the trends were not statistically significant.

**Table 2:** PCB quintiles by diabetes status and age-adjusted PCB levels by diabetes status, sex, and race: Anniston Community Health Survey.

Diabetes status				
Characteristic	Normoglycemia (n = 392)	Prediabetes (n = 169)	Diabetes (n = 205)	Total (n = 766)
PCB quintile (ppb)a								
Q1 (0.11–1.15)		105 (27)		32 (19)		16 (8)		153 (20)
Q2 (1.16–2.42)		82 (21)		27 (16)		44 (22)		153 (20)
Q3 (2.43–4.33)		65 (17)		39 (23)		50 (24)		154 (20)
Q4 (4.34–9.33)		84 (21)		36 (21)		33 (16)		153 (20)
Q5 (9.34–170)		56 (14)		35 (21)		62 (30)		153 (20)
ΣPCBs (ppb)a,b*		6.31 ± 0.58 (2.85)		6.14 ± 0.87 (3.13)		7.71 ± 0.80 (3.62)		6.72 ± 0.44 (3.18)
Sex								
Females**		6.42 ± 0.63 (2.83)		6.31 ± 0.95 (3.09)		7.81 ± 0.83 (3.59)		6.85 ± 0.52 (3.16)
Males		5.99 ± 0.88 (2.91)		5.89 ± 1.02 (3.18)		7.39 ± 1.05 (3.69)		6.42 ± 0.76 (3.24)
Race/ethnicity								
White#		3.45 ± 0.64 (1.88)		2.80 ± 0.92 (1.92)		3.99 ± 0.87 (2.11)		3.41 ± 0.57 (1.97)
Nonwhite		10.30 ± 0.72 (5.09)		9.65 ± 0.93 (5.21)		10.84 ± 0.84 (5.71)		10.26 ± 0.58 (5.33)
Data are n (%) or mean ± SE (GM). aPCB quintiles were derived from the ΣPCBs. bPCB levels were adjusted for age; separate age-adjusted models are presented for diabetes status alone, for diabetes status with sex, and for diabetes status with race/ethnicity. *p = 0.014 for GMs compared across diabetes status. **p = 0.014 for GMs compared across diabetes status with sex in the model; no difference by sex. (p = 0.71). #p < 0.001 for GMs compared by race/ethnicity; no difference across diabetes status. (p = 0.25).								

**Table 3:** PCB levels (log ΣPCBs) by diabetes status, age, and BMI: Anniston Community Health Survey.

Diabetes status										
BMI (kg/m2)	Normoglycemia (n = 392)			Prediabetes (n = 169)			Diabetes (n = 205)			Total (n = 766)		
	< 55 years	≥ 55 years	< 55 years	≥ 55 years	< 55 years	≥ 55 years	< 55 years	≥ 55 years
≤ 24.9		70 (1.13)		57 (6.31)		7 (3.87)		11 (5.23)		7 (4.45)		17 (4.16)		84 (1.40)		85 (5.67)
25–29.9		44 (1.58)		61 (4.90)		17 (3.17)		38 (5.48)		14 (2.41)		29 (7.25)		75 (2.00)		128 (5.54)
30–34.9		51 (1.19)		34 (4.18)		19 (1.45)		33 (5.24)		16 (2.76)		35 (5.44)		86 (1.45)		102 (4.92)
35–39.9		25 (1.27)		14 (5.98)		13 (1.99)		13 (4.29)		17 (2.06)		26 (9.43)		55 (1.64)		53 (6.90)
≥ 40		26 (1.23)		9 (6.01)		11 (1.30)		6 (6.87)		23 (1.87)		21 (7.26)		60 (1.46)		36 (6.86)
Data are [n (GM)]. This GLM model assessed age (< 55, ≥ 55), diabetes status (normoglycemia, prediabetes, diabetes), BMI (five categories above), and log PCB levels. Parameter p-values from the GLM: age, p < 0.001; diabetes status, p < 0.001; BMI, p = 0.33.																

In a separate linear regression model, age, total lipids, total years living in Anniston, and nonwhite race showed significant positive associations with ΣPCBs, whereas we observed a significant inverse association for education. No association was observed for BMI in this model (data not shown).

Results from unadjusted logistic regression modeling indicated a significant increase in the prevalence of diabetes across each quintile of PCB exposure relative to the referent (first quintile); unadjusted ORs (95% CIs) for the second through fifth quintiles were 3.51 (1.84, 6.67), 4.83 (2.53, 9.25), 2.66 (1.37, 5.18), and 7.19 (3.79, 13.63), respectively. After adjustment for standard diabetes-related risk factors (family history, age, BMI, race/ethnicity), sex, total lipids, and lipid-lowering medications, the PCB point estimates for the second through fifth quintiles remained elevated but were similar in magnitude (Table 4). An attenuation in the log-transformed ΣPCB OR also was observed after adjustment for standard factors: The unadjusted OR was 1.65 (95% CI: 1.38, 1.99) per 1 SD increase in log PCBs, whereas the adjusted OR for the PCB–diabetes association was 1.23 (95% CI: 0.88, 1.72).

**Table 4:** Logistic regression models of PCB quintiles and diabetes for the total group and with stratification by age: Anniston Community Health Survey.

Total group (n = 580)			< 55 years (n = 288)			≥ 55 years (n = 292)		
Characteristic	n (diabetes/control)	OR (95% CI)	n (diabetes/control)	OR (95% CI)	n (diabetes/control)	OR (95% CI)
PCB quintile (ppb)												
Q1 (0.11–1.15)		16/102		1.00 (reference)		15/99		1.00 (reference)		1/4		1.00 (reference)
Q2 (1.16–2.42)		44/80		2.18 (0.98, 4.82)		25/46		2.44 (0.91, 6.53)		19/34		4.47 (0.28, 71.38)
Q3 (2.43–4.33)		47/62		2.91 (1.24, 6.83)		17/30		2.29 (0.71, 7.39)		30/32		8.45 (0.53, 134.99)
Q4 (4.34–9.33)		33/79		1.64 (0.64, 4.19)		7/25		1.14 (0.36, 5.52)		26/54		3.74 (0.23, 59.62)
Q5 (9.34–170)		62/55		2.78 (1.00, 7.73)		12/12		4.78 (1.11, 20.6)		50/43		4.19 (0.26, 68.12)
Log PCB per 1 SD increasea		202/378		1.23 (0.88, 1.72)		76/212		1.52 (0.89, 2.58)		126/166		0.93 (0.58, 1.58)
PCB quintiles and the log-transformed PCB variable were derived from the ΣPCBs, wet weight. Individuals with prediabetes were excluded from all models. Covariables were age, sex, BMI (continuous), total lipids, race/ethnicity, family history of diabetes, and taking lipid-lowering medication. Controls are individuals with normoglycemia. aThe same covariables were used in both log-transformed PCB models and quintile models.												

**Figure 1:**
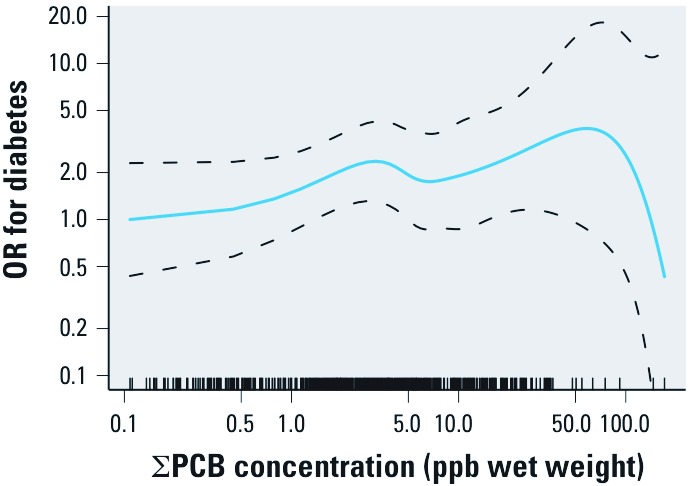
GAM results for serum ΣPCB concentration (ppb wet weight) and OR for diabetes, adjusted for age, sex, BMI (continuous), total lipids, race, family history of diabetes, and taking lipid-lowering medications. The OR referent is the lowest PCB measurement, and dashed lines represent the 95% pointwise confidence band. The tick marks on the *x*-axis represent PCB measurements for each person.

The GAM results in Figure 1 are similar to the quintile results, suggesting increasing diabetes risk with increasing serum PCBs, but possibly via a nonmonotonic relationship, with a decrease between about 3 ppb and 7 ppb. The sharp downtown in dose response at the high end of exposure is based on seven participants with PCB levels > 50 ppb, which precludes precise estimation.

Models stratified by age (< 55 and ≥ 55 years) are shown in Table 4. In the younger participants, after adjustment for established diabetes-related risk factors, the likelihood of diabetes remained significant among those in the highest quintile relative to the lowest (OR = 4.78; 95% CI: 1.11, 20.60). Point estimates in the second and third quintiles also were elevated among the younger participants but to a lesser extent. In the older participants, the ORs were elevated in each of the second through fifth quintiles, but the estimates were imprecise, with wide CIs, because the referent category (first quintile) comprised only five individuals.

Seventy percent of the study participants were female; analyses stratified by sex revealed a positive association between PCBs and diabetes in females and an inverse association in males. This was apparent in both the quintile and the log-transformed ΣPCB models (*p* < 0.05 for sex × PCB interaction terms). In the quintile model, three of four ORs were significantly elevated in females, whereas three of four ORs were < 1.0 in men; each CI included the null value in the analysis of males. The adjusted OR associated with a 1 SD increase in log-transformed PCBs was 1.52 (95% CI: 1.01, 2.28) in females and 0.68 (95% CI: 0.33, 1.41) in males (Table 5).

**Table 5:** Logistic regression [OR (95% CI)] of ΣPCBs, PCB subsets, DDE, and diabetes in the total group and with stratification by sex: Anniston Community Health Survey.

Exposure variable	Total group (n = 580)	Females (n = 427)	Males (n = 153)
PCB quintile (ppb)						
Q1 (0.11–1.15)		1.00 (reference)		1.00 (reference)		1.00 (reference)
Q2 (1.16–2.42)		2.18 (0.98, 4.82)		3.14 (1.25, 7.89)		0.45 (0.08, 2.63)
Q3 (2.43–4.33)		2.91 (1.24, 6.83)		3.26 (1.21, 8.78)		1.08 (0.15, 7.71)
Q4 (4.34–9.33)		1.64 (0.64, 4.19)		2.47 (0.84, 7.30)		0.30 (0.03, 2.76)
Q5 (9.34–170)		2.78 (1.00, 7.73)		4.87 (1.47, 16.12)		0.29 (0.03, 3.09)
ΣPCBs per 1 SD increase		1.21 (0.87, 1.69)		1.52 (1.01, 2.28)		0.68 (0.33, 1.41)
PCB subsets per 1 SD increase						
Mono-ortho		1.18 (0.86, 1.62)		1.47 (0.99, 2.17)		0.74 (0.38, 1.42)
Estrogenic		1.08 (0.80, 1.46)		1.28 (0.89, 1.84)		0.74 (0.40, 1.38)
Di-, tri-, or tetra-ortho		1.20 (0.86, 1.68)		1.50 (1.00, 2.23)		0.68 (0.33, 1.43)
Ryanodine		1.19 (0.85, 1.66)		1.49 (1.00, 2.23)		0.66 (0.32, 1.337
Mono-ortho TEQ		1.20 (0.87, 2.02)		1.51 (1.02, 2.24)		0.71 (0.36, 1.39)
DDE per 1 SD increase		1.22 (0.85, 1.46)		1.42 (1.03, 1.95)		0.61 (0.32, 1.15)
PCB quintiles and the log-transformed PCB variable were derived from the ΣPCBs, wet weight. All congener subsets and DDE were log transformed. Individuals with prediabetes were excluded from all models. Covariables in adjusted models were age, BMI (continuous-PCB quintile models, categorical-log transformed models), total lipids, race/ethnicity, family history of diabetes, and taking lipid-lowering medication. Sex was included in total group models.						

Results from the analyses of log-transformed PCB subsets (e.g., mono-*ortho*, estrogenic), DDE, and diabetes (Table 5) were comparable to results for ΣPCBs in the total group and in the sex-stratified analyses. In females, adjusted ORs were significantly elevated for each congener subset, with the exception of the estrogenic PCBs. ORs were < 1.0 for males and were nonsignificant for all congener subsets; there were 153 males in this analysis compared with 427 females. DDE was associated with a significantly increased log OR of diabetes in women (OR = 1.42; 95% CI: 1.03, 1.95) but not in men (OR = 0.61; 95% CI: 0.32, 1.15), a finding also reported by Turyk et al. (2009a, 2009b).

## Discussion

We found a high prevalence of diabetes in our study population—residents of an area surrounding the oldest former PCB production facility in the United States. Using logistic and GAM modeling, we observed a nonmonotonic increase in the prevalence of diabetes with increasing PCB congener levels in the total group after adjustment for established diabetes risk factors. We also observed a significantly elevated OR in the highest quintile among individuals < 55 years of age. Among individuals ≥ 55 years of age, adjusted ORs were elevated in all quintiles relative to the first quintile; however, the CIs were imprecise because only five participants in this age group had PCB values in the lowest quintile. In the total study group, the adjusted ORs for the PCB subsets and DDE were similar to the ΣPCB estimate. Women had an increased likelihood of diabetes not only for ΣPCB but also for DDE and each subset of PCBs examined, with the exception of the estrogenic subset; men had a decreased likelihood of diabetes based on a much smaller number of observations.

PCB levels in our participants exceeded those observed in the general U.S. population and in previously reported community exposures. Lee et al. (2007) found an increased prevalence of diabetes in NHANES participants 40–59 years of age in the top quarter of the distribution of a mixture of six persistent organic pollutants, including pesticides and dioxins. Although adjusted for most risk factors, their model was not adjusted for family history of diabetes; moreover, individuals with prediabetes were not excluded. For comparison, the top value in the lowest quartile in the present study (1.43 ppb) is within the NHANES third quartile (50th to 75th percentile), whereas the third and fourth quartiles are several times higher (quartile 3 = 3.29–7.42 ppb; quartile 4 = 7.43–171 ppb) (Pavuk et al. 2009). In other studies that evaluated the association of diabetes (Codru et al. 2007; Lee et al. 2006, 2010, 2011a; Turyk et al. 2009a, 2009b; Vasiliu et al. 2006), PCB serum concentrations were mostly less than those found 35 years after production stopped in Anniston. The frequency of diabetes reported in other studies is 5–20%. The estimated weighted prevalence of diabetes in the Anniston population (adjusted for age, race/ethnicity, sex, and oversampling in west Anniston) was 16%, which corresponded to an unweighted prevalence in the study cohort of 27% in 2005–2007. The weighted prevalence for Anniston is comparable to the higher reported values from other PCB and diabetes studies and above the prevalence of self-reported diagnosed diabetes in Alabama in 2005 (9.8%) and in 2008 (11.2%) (CDC 2005, 2008).

In the total group logistic and GAM models, and in participants < 55 years of age, our analyses suggest an increase in the odds of diabetes with increasing PCB levels, although the increase does not appear to be monotonic. Our findings are suggestive of a relatively low-dose PCB effect in the Anniston study population because the ORs increase in the second quintile and remain elevated in all quintiles. Low-dose effects of PCBs were reported in the Coronary Artery Risk Development in Young Adults (CARDIA) study (Lee et al. 2010). In the present study, we observed an association between PCBs and diabetes in younger participants, but not in those ≥ 55 years of age. Our observations in younger Anniston residents are consistent with findings from the CARDIA study in which the PCB–diabetes associations were noted in individuals between 36 and 52 years of age (Lee et al. 2010). The lack of association in older Anniston participants contrasts with findings from the Prospective Investigation of the Vasculature in Uppsala Seniors (PIVUS) study (Lee et al. 2011a), a longitudinal study of elderly in Uppsala, Sweden. The Anniston study group was approximately 50% African American. Other demographic factors and/or differences in access to medical care between Anniston and Uppsala residents likely complicate comparison of the findings across these two populations.

Additional strengths of our study include fasting glucose measurements and the ability to distinguish individuals with prediabetes from the normoglycemic. These participants had characteristics intermediate to those with and without diabetes. For example, 29% of participants classed as having prediabetes self-reported heart disease, compared with 20% of those with normoglycemia and 40% with diabetes. Because of differences in these characteristics, exclusion of participants with prediabetes in the referent group was the physiologically and mechanistically preferable choice.

Our observation that PCBs were positively associated with diabetes in women is similar to that reported for cohorts exposed to PCBs and furans in contaminated rice oil in Taiwan (Wang et al. 2008) and for women exposed to polybrominated biphenyls (PBBs) and PCBs through contaminated milk and animal feed in Michigan (Vasiliu et al. 2006). Results of these studies, however, were not adjusted for family history or prediabetes. Excess diabetes risk in exposed females may be secondary to a higher percentage of body fat, allowing for greater accumulation and retention of lipophilic compounds. Other possible mechanisms include estrogenic activities found in PCB congeners and their metabolites possibly affecting glucose metabolism.

There are several mechanisms that could provide biological plausibility to the observed associations between diabetes and PCBs, dioxins, and other organochlorines, including altered gene transcription, lipid metabolism, insulin production, changes in the insulin signaling pathway (Marchand et al. 2005), and altered glucose transport (Tonack et al. 2007). The association of diabetes and PCBs is also complicated by changes in lipid metabolism caused by diabetes, which may affect the distribution and elimination of lipophilic PCBs, as perhaps suggested by lower PCBs in higher BMI categories in those with diabetes and prediabetes. Because repeated measurements of PCBs or lipids were not performed, these changes (or possible reverse causality) could not be evaluated. Although the slower elimination of dioxin in participants with diabetes was not supported by findings in the Vietnam Veterans study (Michalek et al. 2003), the cross-sectional nature of our study precludes making inferences.

PCBs were produced in Anniston from 1929 to 1971, and this study was undertaken in 2005–2006, at the same time that major remediation began in Anniston. The average duration of residency in Anniston among participants was 28.8 years. Congeners measured in our study participants included those with transient half-lives (e.g., PCB congeners 18, 44, 101, 110, and 151) and those with half-lives of > 15 years (e.g., PCB congeners 118, 138, 170) (Emond et al. 2005; Hansen 2001; Ogura 2004). PCB levels among 1,384 participants measured in 1976 in the Michigan PBB cohort were (from low to high, in quartiles) ≤ 5, 5.1–7.0, 7.1–10.0, and > 10 ppb (Vasiliu et al. 2006). These PCB quartile ranges are similar to those observed in Anniston in 2005, 35 years after PCB production ended; the Anniston quartile ranges, from low to high, were ≤ 1.43, 1.44–3.28, 3.29–7.42, and 7.43–171 ppb. It is likely that the PCB levels in most Anniston residents have decreased since the end of PCB production. In the Michigan PBB cohort, total PCB levels remained stable in 37.5% of participants, increased in 12.2%, and decreased in 50.3% over time in samples collected between 1975 and 1994 (Sweeney et al. 2001).

Households were randomly selected for participation in the present study, as were individuals within households. Although selection bias cannot be ruled out, there were no significant differences by race or sex among those with and without laboratory testing. Older individuals were more likely to have blood testing, as were individuals who were not employed or were retired (*p* < 0.05), an observation that is consistent with less flexible schedules and/or less concern about health among younger participants. Older participants also had higher rates of diabetes, but those were not found to be statistically associated with PCB levels.

Although the ORs (95% CIs) are internally valid as descriptors of associations in our study, they may not represent associations in Anniston as a whole because of our complex sampling design and exclusion of individuals with prediabetes.

## Conclusions

This study demonstrates a statistically significant association of serum PCB levels with increased diabetes prevalence overall, especially among women. Additionally, the most highly exposed individuals < 55 years of age had an elevated risk of diabetes.
